# Novel BODIPY-Loaded Liposomes Enhance Cellular Uptake and PDT Efficacy in 2D and 3D Models

**DOI:** 10.3390/pharmaceutics18080989

**Published:** 2026-08-11

**Authors:** Federica Randisi, Miryam Chiara Malacarne, Francesco Milano, Lucrezia Cappon, Vincenzo De Leo, Emanuela Marras, Davide Odorico, Enrico Caruso, Marzia Bruna Gariboldi

**Affiliations:** 1 Department of Biotechnology and Life Sciences—DBSV, University of Insubria, 21100 Varese, Italy; frandisi1@uninsubria.it (F.R.); miryamcm@hotmail.it (M.C.M.); lcappon@studenti.uninsubria.it (L.C.); emanuela.marras@uninsubria.it (E.M.); dodorico@studenti.uninsubria.it (D.O.); 2 Institute of Sciences of Food Production—CNR-ISPA, 73100 Lecce, Italy; francesco.milano@cnr.it; 3 Department of Chemistry, University of Bari, 70100 Bari, Italy; vincenzo.deleo@uniba.it

**Keywords:** PDT, BODIPY, liposome, uptake, 2D and 3D models

## Abstract

**Background**: Photodynamic therapy (PDT) is a cancer treatment that combines a photosensitizer (PS), light, and oxygen to generate reactive oxygen species (ROS), leading to tumor cell death. PDT efficacy depends largely on PS accumulation within tumors, prompting the development of third-generation PSs and nanotechnology-based delivery systems. Among these, BODIPYs (4,4-difluoro-4-bora-3a,4a-diaza-s-indacene) are promising PSs due to their favorable photophysical properties, while liposomes improve drug delivery, cellular uptake, and sustained release profiles. This study describes the synthesis of two novel BODIPY derivatives differing in the position of a methyl ester group on the meso-phenyl ring, their incorporation into liposomes, and evaluation of PDT efficacy. **Methods**: Cellular uptake of BODIPY-loaded liposomes, intracellular ROS generation, apoptosis, necrosis, and lipid peroxidation were assessed by flow cytometry in colorectal and ovarian cancer cell lines. The antitumor activity of the liposomal formulations was further evaluated in both 2D and 3D models using MTT and clonogenic assays. The involvement of ferroptosis and necroptosis in PDT-induced cell death was also investigated. **Results**: Liposomal formulations significantly enhanced cellular uptake compared with free compounds. Following light activation, both formulations induced potent antitumor effects through multiple cell death mechanisms, including canonical and non-canonical pathways, and maintained strong efficacy in 3D tumor spheroids. **Conclusions**: Liposome-encapsulated BODIPYs represent promising PDT agents by improving cellular uptake and eliciting robust antitumor activity through complementary cell death mechanisms. Furthermore, the methyl ester substituent on the meso-phenyl ring provides a versatile platform for future conjugation with targeting ligands, supporting the development of third-generation, tumor-targeted photosensitizers and warranting further preclinical investigation.

## 1. Introduction

Photodynamic therapy (PDT) is an attractive, minimally invasive therapeutic approach for the treatment of cancer and other pathological conditions [1,2]. The technique combines a photosensitizer (PS), light of an appropriate wavelength, and molecular oxygen to generate reactive oxygen species (ROS), particularly singlet oxygen (^1^O_2_), which induce oxidative damage and ultimately cell death [3,4]. Despite the promising clinical outcomes achieved with PDT, its therapeutic efficacy remains limited and strongly dependent on the physicochemical and biological properties of the photosensitizer [5].

Among the factors governing PDT success, efficient cellular uptake and intracellular accumulation of the PS play a pivotal role [6,7,8]. Since ROS possess extremely short lifetimes and diffusion distances, photodynamic damage occurs primarily in the immediate vicinity of the activated photosensitizer. Consequently, insufficient cellular internalization can severely limit ROS generation at relevant intracellular targets, reducing treatment efficacy [9]. Moreover, the intracellular distribution of the PS influences the activation of specific cell death pathways, affecting both the magnitude and mechanism of the therapeutic response [10]. Therefore, strategies to enhance PS delivery to target cells are critical to developing more effective PDT protocols [11,12,13,14,15].

In addition to inefficient cellular internalization, many photosensitizers suffer from intrinsic limitations, including poor aqueous solubility, low bioavailability, and aggregation in biological media [4,16]. These drawbacks often result in reduced photodynamic activity and hinder their clinical translation. Nanocarrier-based delivery systems have been extensively investigated to overcome such limitations and improve the pharmacological profile of photosensitizers [3,12,17]. Among them, liposomes have attracted considerable attention owing to their excellent biocompatibility, biodegradability, low toxicity, and ability to encapsulate both hydrophilic and hydrophobic compounds. Furthermore, liposomal formulations can protect photosensitizers from degradation, improve their dispersion in aqueous environments, and promote cellular uptake through endocytic mechanisms [18,19,20].

Boron-dipyrromethene (BODIPY) derivatives have recently emerged as a versatile class of photosensitizers for PDT, owing to their favorable photophysical properties, including strong absorption coefficients, high photostability, tunable optical characteristics, and efficient ROS generation following appropriate structural modification, making them attractive candidates for phototherapeutic applications [21,22,23,24]. Although BODIPY photosensitizers are not yet clinically approved, their photophysical profile compares favorably with that of established photosensitizers such as porphyrins, chlorins, and phthalocyanines [1,25]. Clinically established porphyrins, exemplified by Photofrin^®^, are highly effective but generally exhibit relatively low molar absorption coefficients within the therapeutic window and prolonged cutaneous photosensitivity due to slow systemic clearance. Chlorins and phthalocyanines exhibit stronger absorption in the red/near-infrared region, allowing deeper tissue penetration [1,26]. BODIPY derivatives are not inherently exempt from these limitations: like many photosensitizers, they are often poorly soluble in water and can be prone to aggregation. However, their high degree of structural tunability allows for targeted modifications, such as steric substitution at the core, which can suppress aggregation while preserving high molar extinction coefficients [1,21,22,24,25]. Incorporation within liposomal carriers may represent an effective strategy to overcome this limited aqueous solubility and enhance cellular accumulation, maximizing their delivery and photodynamic potential [27]. Nevertheless, despite these promising characteristics, BODIPY derivatives still lack extensive in vivo and clinical validation.

In addition to optimizing PS delivery, the evaluation of PDT efficacy requires experimental models that adequately reproduce the complexity of the tumor microenvironment. While conventional two-dimensional (2D) cell cultures remain valuable for preliminary investigations, three-dimensional (3D) tumor spheroids more closely mimic in vivo conditions, including cell–cell interactions, nutrient and oxygen gradients, and diffusion barriers that can affect photosensitizer penetration and therapeutic response [28,29,30]. Consequently, the combined assessment of PS uptake and PDT efficacy in both 2D and 3D models provides a more comprehensive evaluation of the therapeutic potential of novel formulations [31,32,33].

In the present study, two novel BODIPY derivatives differing in the position of a methyl ester group on the meso-phenyl ring were designed and synthesized. Both compounds were incorporated into liposomes, and the ability of this formulation to enhance cellular delivery and improve photodynamic efficacy was evaluated in both 2D monolayer cultures and 3D spheroid models. The human colorectal carcinoma cell line HCT116 and the human ovarian adenocarcinoma cell line SKOV3 were selected as representative in vitro models of two clinically relevant solid tumors that remain major causes of cancer-related mortality, particularly in advanced or recurrent disease, where alternative therapeutic approaches such as PDT are being actively explored [34,35,36]. HCT116 is a well-established colorectal cancer model widely employed for evaluating PDT-mediated anticancer strategies [20,36,37], whereas SKOV3 is extensively used to investigate novel therapeutic approaches for ovarian cancer, including PDT, owing to its aggressive phenotype and intrinsic chemoresistance [34,38]. Notably, these two cell lines have previously been used together to evaluate new BODIPY and aza-BODIPY photosensitizers, facilitating direct comparisons with previous studies and supporting the translational relevance of the present work [39,40].

The biological activity of the liposomal formulations was comprehensively characterized by assessing cell viability, ROS generation, and the induction of apoptotic, necrotic, and ferroptotic cell death in both cellular models.

## 2. Materials and Methods

### 2.1. General Remarks

^1^H NMR, UV-Vis absorption, fluorescence emission, HPLC, and MS analysis were performed as previously reported by our group [35,41].

Methyl 3-formylbenzoate, methyl 4-formylbenzoate, 2,4-dimethylpyrrole, 2,3-dichloro-5,6-dicyano-1,4-benzoquinone (DDQ), triethylamine (Et_3_N), boron trifluoride diethyl etherate (BF_3_·Et_2_O), iodine (I_2_), iodic acid (HIO_3_), trifluoroacetic acid (TFA), and cholesterol (>99%) were purchased as commercial reagents from Sigma-Aldrich (Merck Life Sciences S.r.l., Milan, Italy) and used without further purification. All cell culture media and reagents, unless otherwise specified, were purchased from Euroclone (Milan, Italy).

Dichloromethane (CH_2_Cl_2_) used in the synthesis of BODIPY compounds was dried by distillation over CaCl_2_ and collected directly into the two-necked flask in which the reaction was performed.

Thin-layer chromatography (TLC) was performed on precoated silica gel 60 F254 plates (0.2 mm thickness). Column chromatography was carried out using silica gel 60 (70–230 mesh, Merck Life Sciences S.r.l., Milan, Italy) as the stationary phase.

### 2.2. BODIPY Synthesis and Characterization

#### 2.2.1. 4,4-Difluoro-8-(3-(methoxycarbonyl)phenyl)-1,3,5,7-tetramethyl-4-bora-3a,4a-diaza-s-indacene (**1**)

In a previously oven-dried two-neck round-bottom flask, 2,4-dimethylpyrrole (6.6 mmol, 679 μL; MW = 95.14 g/mol; d = 0.924 g/mL) and methyl 3-formylbenzoate (3.0 mmol, 490 mg; MW = 164.16 g/mol) were dissolved in 90 mL of anhydrous THF under a nitrogen atmosphere. Trifluoroacetic acid (TFA, 10 drops) was added, and the reaction mixture was stirred at room temperature until complete consumption of the aldehyde, as monitored by TLC using CH_2_Cl_2_ as the eluent (typically ~24 h). Subsequently, DDQ (3.0 mmol, 680 mg; MW = 227 g/mol) was added, and the mixture was allowed to stir for an additional 4 h. The reaction flask was then cooled in an ice bath, after which triethylamine (18 mL) was added dropwise, followed by the slow addition of boron trifluoride diethyl etherate (18 mL). The reaction was allowed to proceed under stirring overnight. Progress of the reaction was monitored by TLC (CH_2_Cl_2_).

Upon completion, the reaction mixture was washed twice with 1 M aqueous HCl solution and repeatedly with water. The organic phase was dried over anhydrous Na_2_SO_4_, filtered, and the solvent was removed under reduced pressure using a rotary evaporator. The crude product was purified by silica gel column chromatography, employing a hexane/CH_2_Cl_2_ eluent system, initially in a 9:1 ratio and subsequently in a 7:3 ratio. This procedure afforded 450 mg of the target compound as a purified product, corresponding to a yield of 39.2%.

C_21_H_21_BF_2_N_2_O_2_ M.W.: 382.21 g/mol

^1^H NMR (CDCl_3_): 8.20 (m, 1H); 7.62 (m, 1H); 7.52 (d, 1H); 7.43 (d, 1H); 6.01 (s, 2H); 3.99 (s, 3H); 2.58 (s, 6H); 1.37 (s, 6H) UV-Vis (DCM): 504 nm (ε = 53,700) UV-Vis (MeOH): 500 nm (ε = 69,700). Φ_fl_: (DCM) 516 nm = 0.48.

#### 2.2.2. 4,4-Difluoro-8-(4-(methoxycarbonyl)phenyl)-1,3,5,7-tetramethyl-4-bora-3a,4a-diaza-s-indacene (**2**)

With the same procedure reported for **1**, 2,4-dimethylpyrrole (6.6 mmol, 679 μL; MW = 95.14 g mol^−1^; d = 0.924 g mL^−1^) and methyl 4-formylbenzoate (3.0 mmol, 490 mg; MW = 164.16 g mol^−1^) were reacted, affording the desired product as a solid (340 mg, 29.6% yield).

C_21_H_21_BF_2_N_2_O_2_ M.W.: 382.21 g/mol

^1^H NMR (CDCl_3_): 8.20 (d, 2H); 7.42 (d, 2H); 6.01 (s, 2H); 3.99 (s, 3H); 2.58 (s, 6H); 1.38 (s, 6H) UV-Vis (DCM): 502 nm (ε = 49,400) UV-Vis (MeOH): 500 nm (ε = 66,900). Φ_fl_: (DCM) 515 nm = 0.45.

#### 2.2.3. 4,4-Difluoro-2,6-diiodo-8-(3-(methoxycarbonyl)phenyl)-1,3,5,7-tetramethyl-4-bora-3a,4a-diaza-s-indacene (**1I**) and 4,4-Difluoro-2,6-diiodo-8-(4-(methoxycarbonyl)phenyl)-1,3,5,7-tetramethyl-4-bora-3a,4a-diaza-s-indacene (**2I**)

Compound **1** (200 mg, 0.523 mmol; MW = 382.22 g/mol) and compound **2** (200 mg, 0.523 mmol; MW = 382.22 g·mol^−1^) were transferred into a 100 mL round-bottom flask. Iodine (332 mg, 1.31 mmol; MW = 254 g/mol) and iodic acid (230 mg, 1.31 mmol; MW = 176 g/mol) were dissolved in 25 mL of ethanol and added to the reaction flask. The resulting mixture was stirred at room temperature overnight.

After completion of the reaction, the crude mixture was first washed with water, then extracted with dichloromethane. The organic phase was subsequently washed with an aqueous sodium bisulfite solution (NaHSO_3_) to remove residual iodine. The organic phase was separated, dried over anhydrous Na_2_SO_4_, filtered, and concentrated under reduced pressure using a rotary evaporator.

Purification by silica gel column chromatography, using a hexane/CH_2_Cl_2_ (1:1) eluent system, afforded the desired product, corresponding to a yield of 57.3% (190 mg) for **1I** and 66.3% (220 mg) for **2I**.

**1I**: C_21_H_19_BF_2_I_2_N_2_O_2_ M.W.: 634.00 g/mol

^1^H NMR (CDCl_3_): 8.24 (m, 1H); 7.66 (m, 1H); 7.50 (d, 1H); 7.40 (d, 1H); 4.01 (s, 3H); 2.67 (s, 6H); 1.38 (s, 6H) UV-Vis (DCM): 538 nm (ε = 47,100) UV-Vis (MeOH): 534 nm (ε = 51,800). Φ_fl_: (DCM) 551 nm = 0.02. HPLC: retention time = 12′45″ (99.0%), MS (ESI): M^+^ found: 639.98.

**2I**: C_21_H_19_BF_2_I_2_N_2_O_2_ M.W.: 634.00 g/mol

^1^H NMR (CDCl_3_): 8.23 (d, 2H); 7.40 (d, 2H); 4.01 (s, 3H); 2.67 (s, 6H); 1.39 (s, 6H) UV-Vis (DCM): 538 nm (ε = 53,300) UV-Vis (MeOH): 534 nm (ε= 73,500). Φ_fl_: (DCM) 556 nm = 0.01. HPLC: retention time = 13′10″ (98.0%), MS (ESI): M^+^ found: 639.97.

### 2.3. Incorporation of the BODIPYs into Liposomes and Characterization

The lipid components, namely Lipoid S100 (LS100, 213 μL of a 100 mg·mL^−1^ solution in CHCl_3_), PEG-2000-PE (Lipoid, 117 μL of a 30 mg·mL^−1^ solution in CHCl_3_), and cholesterol (28 μL of a 40 mg·mL^−1^ solution in CHCl_3_), were combined with solutions of iodinated and non-iodinated BODIPY dyes in dichloromethane: iodinated (225 μL, 2 mM) and non-iodinated (90 μL, 0.5 mM). The resulting molar ratio of lipid components and dyes was 85:4:9.5:1.5 (LS100:PEG-2000-PE:cholesterol:BODIPY).

The mixture was transferred to a 10 mL round-bottom flask, and the organic solvents were removed under reduced pressure using a rotary evaporator until a thin, homogeneous lipid film formed on the inner wall of the flask. The dried film was hydrated with phosphate-buffered saline (PBS, 4.5 mL), yielding a suspension of multilamellar vesicles with a total lipid concentration of 5.8 mg·mL^−1^, corresponding to final concentrations of 100 μM iodinated BODIPY and 10 μM non-iodinated BODIPY.

The suspension was then subjected to 11 extrusion cycles using a LiposoFast extrusion system (Avestin, Ottawa, ON, Canada) equipped with 100 nm polycarbonate membranes, affording unilamellar liposomes with an average diameter of approximately 100 nm.

#### Dynamic Light Scattering

The hydrodynamic diameter, polydispersity index (PDI), and zeta potential of the liposomal formulations, namely **L1** and **L2**, were determined by dynamic light scattering (DLS) using a Malvern Zetasizer Nano ZS (Malvern Panalytical, Malvern, UK) equipped with a folded capillary cell. Measurements were performed at 25 °C with automatic data acquisition, and particle size distributions were calculated from the intensity-weighted autocorrelation function. All measurements were performed in triplicate.

### 2.4. Photobleaching and Single Oxygen Production

Photostability analysis was performed to evaluate the absorbance decay of each photosensitizer in the liposomal formulation under green LED irradiation. The PSs, dissolved in DMSO at a concentration of 1 mM, were diluted in Milli-Q water to a final concentration of 1 × 10^−5^ M and transferred to a quartz cuvette. Samples were irradiated with green LED light for 1 h. During irradiation, UV-Vis absorbance spectra were recorded every 20 min using a spectrophotometer, and the absorbance at the maximum absorption wavelength (λ_max) of each PS was monitored over time.

The singlet oxygen (^1^O_2_) generation capacity of the investigated molecules was assessed by monitoring the photooxidative degradation of 1,3-diphenylisobenzofuran (DPBF), a widely used chemical probe and efficient ^1^O_2_ scavenger. Singlet oxygen production was determined by measuring the decrease in DPBF absorbance at 410 nm by UV-Vis spectrophotometry. Briefly, a solution of 8 mL of isopropanol, 50 μM of 1,3-diphenylisobenzofuran (DPBF), and 2.5 μM of PS is prepared. A total of 2 mL of this solution is transferred to a quartz cuvette and irradiated from above for 10 min with a green LED. Absorbance measurements at 410 nm were acquired throughout irradiation to monitor DPBF degradation kinetics in real time. The rate of DPBF photodegradation was used as an indirect measure of singlet oxygen production and was normalized to that obtained using Rose Bengal, a well-established singlet oxygen producer, used as a reference standard under identical experimental conditions [42].

### 2.5. Biological Studies

#### 2.5.1. Cell Lines and In Vitro Culture Conditions

All the cell lines were originally obtained from ATCC (American Type Culture Collection, Manassas, VA, USA) and maintained under standard culture conditions (37 °C; 5% CO_2_). HCT116 (human colon cancer) cells were maintained in DMEM medium, while SKOV3 (human ovarian cancer) cells were cultured in DMEM-F12 medium. All media were supplemented with 10% fetal calf serum, 1% glutamine, and 1% antibiotic mixture; an extra 1% sodium pyruvate and 1% non-essential amino acids were added to DMEM.

To produce the corresponding spheroids, HCT116 cells were detached, and 5 × 10^3^ cells/well were then seeded onto Nunclon Sphera 96U plates (Thermo, Milan, Italy) and incubated at 37 °C in a 5% CO_2_ atmosphere. Spheroids were used on day 4 after seeding.

#### 2.5.2. Intracellular Accumulation of Free and Liposome-Loaded BODIPYs and Diffusion of BODIPY-Loaded Liposomes Inside Spheroids

Intracellular accumulation of the different formulations was evaluated in HCT116 and SKOV3 cell lines following 24 h of incubation with the non-iodinated compounds (100 nM) through cytofluorimetric analysis, exploiting their intrinsic fluorescence. Free **1** and **2** BODIPYs were diluted in DMSO at the final concentration of 100 nM. At the end of the exposure time, treated cells were detached using a trypsin-EDTA solution, washed in ice-cold PBS, resuspended in PBS, and analyzed with a FACSCalibur flow cytometer (Becton Dickinson, Mountain View, CA, USA). Data were processed using CellQuestPRO 5.1 software (Becton Dickinson).

Spheroids were obtained as reported and incubated with free (**1** and **2**) or liposome-loaded BODIPYs (**L1** and **L2**). After 24 h, fluorescence distribution into spheroids was evaluated by confocal microscopy. Spheroids were transferred from 96-well plates to a microscope slide, washed with HBSS, and directly observed under a Leica Stellaris 5 confocal microscope (Leica Biosystems, Buccinasco (MI), Italy). Images of the equatorial plane of the spheroids were acquired and analyzed using LasX office 1.4.8982 software by randomly drawing 12 radial lines on the equatorial plane image and recording the fluorescence at each pixel.

#### 2.5.3. Photodynamic Effects in 2D and 3D Models

The phototoxic activity of **L1** and **L2** was assessed using the MTT assay (3-(4,5-dimethylthiazol-2-yl)-2,5-diphenyltetrazolium bromide), according to previously reported procedures [20]. Briefly, cells were seeded in 96-well plates at a density of 3 × 10^4^ cells/mL and allowed to adhere and proliferate for 48 h before treatment with the PSs at the indicated concentrations (0.1–50 nM). Following a 24 h incubation period, the PS-containing medium was removed and replaced with fresh phosphate-buffered saline (PBS). Cells were subsequently irradiated for 1 h using a green LED source with an irradiance of 3.04 × 10^−3^ W·cm^−2^, corresponding to a total fluence of 10.9 J/cm^2^. After irradiation, PBS was replaced with drug-free culture medium, and the cells were incubated for an additional 24 h in the dark at 37 °C. Cell viability was then determined by the MTT assay, and absorbance was measured at 570 nm using an iMark Reader (Bio-Rad Instruments, Segrate (MI), Italy). Untreated control cells were subjected to the same experimental procedure, including light exposure, but without PS treatment. Cytotoxicity was quantified by calculating IC_50_ values from dose–response curves generated by non-linear regression analysis using CalcuSyn 2.0 software (Biosoft, Cambridge, UK).

To assess light-independent cytotoxicity, parallel dark-control experiments were performed in which PS-treated cells were maintained under identical conditions but shielded from light exposure throughout the experiment.

The long-term reproductive capacity of HCT116 cells following PDT with **L1** and **L2** was assessed by clonogenic assays. Briefly, cells were seeded into 6-well plates and allowed to adhere and proliferate for 48 h before being exposed to **L1** and **L2** for 24 h. Subsequently, cells were irradiated for 1 h under the previously described experimental conditions, detached, and reseeded at a density of 250 cells per well in duplicate in 6-well plates. Cells were then incubated at 37 °C for 7–10 days to allow colony formation. At the end of the incubation period, colonies were fixed with 100% methanol, washed with PBS, and stained with methylene blue. Following a final PBS wash, colonies consisting of at least 50 cells were counted to determine the clonogenic survival fraction.

The phototoxic effects of the investigated compounds were further investigated in HCT116 multicellular spheroids by monitoring spheroid growth through a dye exclusion assay. Spheroids were treated with concentrations of **L1** and **L2** corresponding to the IC_50_ values previously determined in 2D-cultured HCT116 cells. After 24 h of incubation, the drug-containing medium was removed, and the spheroids were irradiated for 1 h in PBS under the same conditions described above. Following light exposure, spheroids were maintained in drug-free culture medium for 72 h. At 24 h intervals, three to five spheroids from each treatment group were independently collected and enzymatically dissociated using a trypsin–EDTA solution. Cell viability was determined by Trypan Blue exclusion, and viable cells were counted using a Bürker hemocytometer (Merck Life Sciences S.r.l., Milan, Italy). Control spheroids received culture medium alone and were subjected to the same incubation and irradiation procedures as the PS-treated samples. Representative images of spheroids were acquired immediately before treatment (t_0_) and every 24 h, up to 72 h, using a camera connected to an Olympus IX81 microscope (Olympus, Segrate (MI), Italy) equipped with a digital camera.

#### 2.5.4. Evaluation of Intracellular Levels of ROS

The intracellular generation of ROS following treatment with the PSs was evaluated by exploiting the fluorescence derived from the reaction between 2,7-dichlorodihydrofluoresceindiacetate (H2DCF-DA). H2DCF-DA easily diffuses into cells, where it is hydrolyzed by intracellular esterases and oxidized by ROS to 2′,7′-dichlorofluorescein (DCF), an impermeable, highly fluorescent compound. Thus, the fluorescence generated is directly proportional to ROS levels.

For the analyses, cells were seeded in 12-well plates (6 × 10^4^ cells/well) and treated 48 h later with equitoxic concentrations of the formulations corresponding to their respective IC_50_ values. Following 24 h of incubation, cells were detached, washed in PBS, resuspended in an H2DCF-DA (10 µM) in PBS, and incubated at 37 °C in the dark for 45 min. After this period, samples were irradiated for 5 min, and fluorescein fluorescence was measured by a FACSCalibur flow cytometer through a 530 nm or 575 nm band-pass filter, and intracellular ROS generation was quantified in arbitrary units based on the median fluorescence intensity (MFI) using CellQuestPRO 5.1 software. Positive controls were also included, in which 3 µL of 30% hydrogen peroxide was added to a control sample.

#### 2.5.5. Evaluation of PDT-Induced Cell Death

The percentages of apoptotic and necrotic cells were evaluated by flow cytometric analysis following staining with propidium iodide. Cells were seeded in 6-well plates (HCT116: 2 × 10^5^/well; SKOV3: 3 × 10^5^/well) and allowed to attach and grow for 48 h in a CO_2_ incubator at 37 °C before treatment with the compounds at their respective IC_50_ values. After 24 h, cells were irradiated and incubated for 24 h in the dark at 37 °C in drug-free medium. To assess the percentage of apoptotic cell death, at the end of treatment, cells were harvested, washed in PBS, and fixed in 70% ethanol at −20 °C for at least 45 min. After a further wash in PBS, DNA was stained with a solution of PI/RNAse (50 µg/mL/30 U/mL) in PBS at room temperature for 15 min. Samples were then analyzed through a FACScalibur Becton Dickinson flow cytometer equipped with an air-cooled argon ion laser (15 mW, 488 nm), using CellQuestPRO 5.1 software. The fluorescent emission of PI was collected through a 575 nm band-pass filter, and the percentage of apoptotic cells in each sample was determined based on the sub-G1 peaks detected in monoparametric histograms acquired in log mode. Evaluation of apoptotic cells from spheroids was performed following the same protocol after spheroid disaggregation.

Necrotic cells were detected by omitting the fixation step from the previously described staining protocol. Under these conditions, PI selectively penetrates cells with compromised plasma membrane integrity, thereby enabling the detection of necrotic cells.

To further investigate the contribution of necroptosis to cell death induced by **L1** and **L2**, MTT assays were performed in the presence of Necrostatin-1 (Nec-1, 20 μM), a well-established RIPK-1 inhibitor.

The involvement of ferroptosis in the mechanism of cell death was investigated by performing MTT assays in the presence of Ferrostatin-1 (1 μM), a well-established inhibitor of ferroptosis. In addition, lipid peroxidation was evaluated following treatment and PDT, using BODIPY 581/591 C11 as a fluorescent probe, according to previously reported procedures [43].

#### 2.5.6. Statistical Analysis

Statistical analyses were performed using GraphPad Prism (version 8.4). Unless otherwise specified, all experiments were conducted with at least three independent biological replicates. Data normality was assessed using the Shapiro–Wilk test. For IC_50_ values, normality assumptions were verified on log-transformed concentration values. Depending on the experimental design, statistical comparisons were performed using one- or two-way ANOVA, followed by Bonferroni’s post hoc test for multiple comparisons. Differences were considered statistically significant at *p* < 0.05.

## 3. Results

### 3.1. Free and Liposome-Loaded BODIPY Characterization

The synthesis of the two BODIPY derivatives was carried out according to the procedures reported by Akkaya and Liu [44,45]. The synthetic route involved the condensation of two units of 2,4-dimethylpyrrole with an aromatic aldehyde (in this case, methyl 3- or 4-formylbenzoate), thereby providing the meso-methine bridge linking the two pyrrole units and affording the corresponding dipyrromethane intermediate. This condensation was performed in THF under a nitrogen atmosphere using acid catalysis (TFA). The resulting intermediate was subsequently oxidized with DDQ, then complexed with BF_3_·OEt_2_ in the presence of Et_3_N, yielding the corresponding BODIPY derivatives. Purification by liquid–liquid extraction and silica gel column chromatography afforded **1** and **2** in isolated yields of 39.2% and 29.6%, respectively.

The introduction of heavy atoms into the BODIPY scaffold represents a well-established strategy to enhance its suitability as a photosensitizer for photodynamic therapy (PDT). In particular, the incorporation of iodine atoms at the β-positions of the BODIPY core promotes the heavy-atom effect, which enhances spin–orbit coupling, suppresses fluorescence emission, and increases intersystem crossing to the triplet excited state, thereby improving singlet oxygen generation efficiency [46].

**Figure 1:**
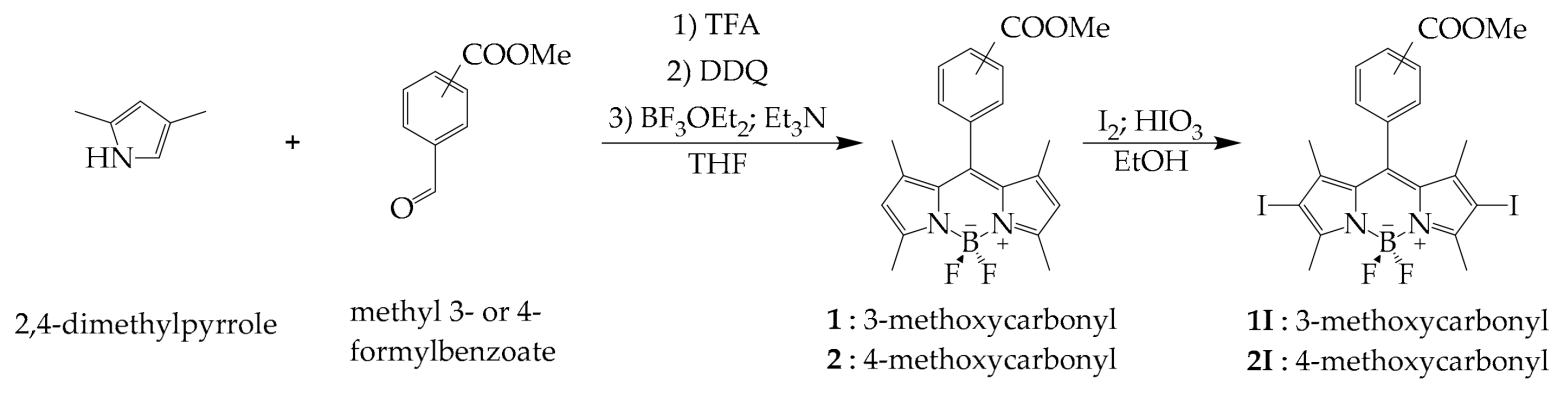
Synthesis scheme for the preparation of compounds **1** and **2** and their iodinated derivatives **1I** and **2I**.

The iodination of **1** and **2** was performed following a modified version of the Nagano protocol previously developed in our laboratory [47]. The reaction was carried out at room temperature overnight using I_2_ and HIO_3_ in ethanol as the iodinating system. After purification by silica gel column chromatography, the corresponding diiodinated derivatives, **1I** and **2I**, were obtained in good isolated yields of 57.3% and 66.3%, respectively (Figure 1).

The synthesized compounds were characterized by fluorescence quantum yield and relative lipophilicity. As expected, iodination markedly reduced the fluorescence quantum yield due to the heavy-atom effect, which enhances intersystem crossing at the expense of fluorescence emission [27,48]. This trend is consistent with prior reports showing that iodinated or halogenated BODIPY derivatives exhibit strongly quenched fluorescence relative to their non-halogenated counterparts [49,50]. Specifically, the non-iodinated precursors **1** and **2** exhibited fluorescence quantum yields of 0.48 and 0.45, respectively, whereas the corresponding iodinated derivatives **1I** and **2I** showed values of 0.02 and 0.01, which fall within the range reported for iodinated BODIPY photosensitizers in the literature [50,51]. Both the non-iodinated and iodinated derivatives exhibited Stokes shifts of approximately 10 nm.

The relative lipophilicity of **1I** and **2I** was assessed by reversed-phase HPLC on a C18 column. Although this approach does not provide absolute lipophilicity values, it is widely used to enable reliable relative comparison among structurally related compounds analyzed under identical chromatographic conditions [52,53]. Compound **1I**, bearing the methoxycarbonyl substituent in the *meta* position, eluted earlier than **2I** (*t*_R_ = 12 min 25 s vs. 13 min 10 s), indicating slightly lower lipophilicity. This isomerism in aromatic systems can measurably affect apparent lipophilicity, and substitution at the *meta* position can reduce logKw relative to related analogs [54,55]. This is also in line with BODIPY-focused lipophilicity studies showing that substituent identity and placement alter dye lipophilicity in predictable ways [53].

**1I** and **2I** were incorporated into liposomes prepared by the thin-film hydration method followed by extrusion. The lipid composition, Lipoid S100 (LS100)/cholesterol/PEG-2000-PE at 85:9.5:4 mol%, was selected to combine complementary functional roles: LS100, a natural soybean phosphatidylcholine with high phosphatidylcholine content (≥94%), constitutes the main bilayer matrix and confers membrane fluidity; cholesterol modulates bilayer rigidity and structural stability; and PEG-2000-PE provides a polyethylene glycol surface coating that reduces inter-vesicle aggregation and prolongs colloidal stability in suspension. Each formulation also incorporated the corresponding non-iodinated BODIPY analog (**1** or **2**) at a lower molar fraction, exploiting its intrinsic fluorescence as a tracer for intracellular localization and bioimaging studies. The final lipid film was hydrated with PBS and extruded through 100 nm polycarbonate membranes to yield unilamellar vesicles, which were subsequently sterilized by filtration through 0.22 µm membranes.

The planar and compact geometry of the BODIPY core, together with its pronounced lipophilic character, further enhanced by the hydrophobic meso-phenyl substituent, and typically requiring appropriate delivery strategies in aqueous media, promotes spontaneous and efficient insertion into the phospholipid bilayer. Consistent with this structural rationale, all formulations achieved loading efficiencies above 80%, with the modest residual variability reflecting operator-dependent losses during thin-film formation and hydration rather than inherent physicochemical limitations. The precise depth and orientation of the chromophore within the bilayer (e.g., through fluorescence quenching assays or molecular dynamics simulations) were not investigated in the present study.

The physicochemical properties of the resulting formulations, including hydrodynamic size, surface charge, and optical behavior, were assessed by DLS and UV-Vis spectroscopy. Since the meta/para position of the methoxycarbonyl substituent does not affect the spectroscopic properties of the BODIPY core, and since the liposomal composition and preparation method were identical for both formulations, full characterization is reported for **L1** only, being representative of both formulations. As shown in Table 1, all formulations displayed a hydrodynamic diameter close to the theoretical value of 100 nm, with low PDI values (<0.15) indicating a narrow and homogeneous size distribution. The zeta potential was slightly negative in all cases (approximately −10 to −13 mV), likely reflecting, at least in part, the anionic character of the PEG-2000-PE component, with a possible additional contribution of minor anionic components naturally present in the LS100 batch (e.g., phosphatidic acid, phosphatidylinositol) [56,57,58]. The moderate magnitude of the measured values is consistent with the “hidden charge effect” described for PEGylated vesicles, whereby the PEG layer displaces the electrokinetic shear plane further from the membrane surface, causing the measured zeta potential to underestimate the true surface potential [59]. Liposome colloidal stability is primarily ensured by the steric hindrance provided by the PEG-2000-PE coating, while the mild negative surface charge likely provides an additional contribution [57,58,60].

**Figure 2:**
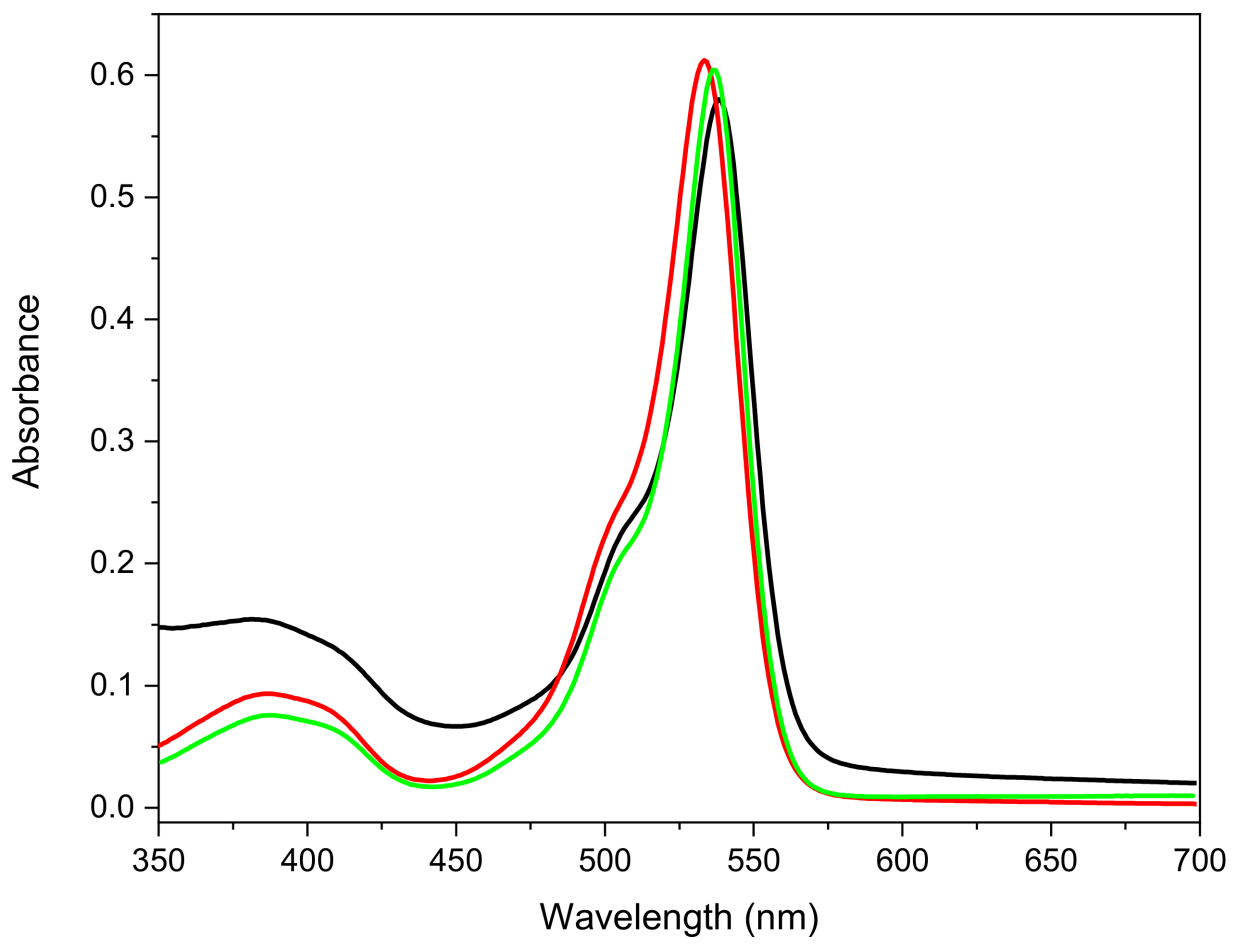
UV-Vis absorption spectra of **L1** diluted 1:10 in Milli-Q water (black) and methanol (red) and of the free compound **1I** in CH_2_Cl_2_ normalized to the methanol spectrum (green).

The absence of BODIPY aggregation within the liposomal formulations was confirmed by UV-Vis spectroscopy. Liposomes were diluted 1:10 in either Milli-Q water or methanol; in the latter, complete dissolution of the vesicles allowed acquisition of aggregate-free reference spectra. The spectrum of **1I** in CH_2_Cl_2_, normalized to the methanol spectrum, was included as an organic solvent reference. As shown in Figure 2, the absorption peak of the BODIPY chromophore retained its shape and position (~535 nm) in all conditions, and the vibrational shoulder at ~500 nm was equally well-resolved in intact liposomes, methanol-dissolved liposomes, and the organic solvent reference, with no broadening or hypochromic shift detectable in the intact liposomal suspensions, spectral signatures that would be expected in the presence of H- or J-type aggregates, respectively.

These findings indicate the absence of significant H- or J-type aggregation of the BODIPY within the bilayer. This behavior can be rationalized on structural grounds: the four methyl substituents at positions 1, 3, 5, and 7 of the BODIPY core create steric hindrance that prevents both face-to-face π-stacking (H-aggregates) and slipped-stack arrangements (J-aggregates). Additionally, the steric clash between the methyl groups at positions 1 and 7 and the ortho hydrogens of the meso-phenyl ring enforces a nearly perpendicular orientation of the aryl substituent relative to the BODIPY core. This geometry disrupts molecular planarity, limits π–π stacking interactions, and thereby reduces the propensity for ordered intermolecular aggregation. Although the combined spectroscopic and structural evidence argues against significant H- or J-type aggregation, direct biophysical confirmation of a fully molecularly dispersed state (e.g., by fluorescence lifetime or anisotropy measurements) was beyond the scope of the present study. The slight increase in baseline absorbance at shorter wavelengths observed in aqueous suspensions is attributable to light scattering from the colloidal particles and does not reflect any alteration of the chromophore.

To assess the photostability of the synthesized BODIPY derivatives, the free compounds and their liposomal formulations were irradiated with green LED light for 1 h. Changes in their absorption spectra were monitored by UV-Vis spectroscopy throughout the irradiation period. In addition, spectral profile variations, including the appearance of new absorption bands or shifts in the absorption maxima, were evaluated as potential indicators of photoproduct formation or structural modification of the photosensitizers.

**Figure 3:**
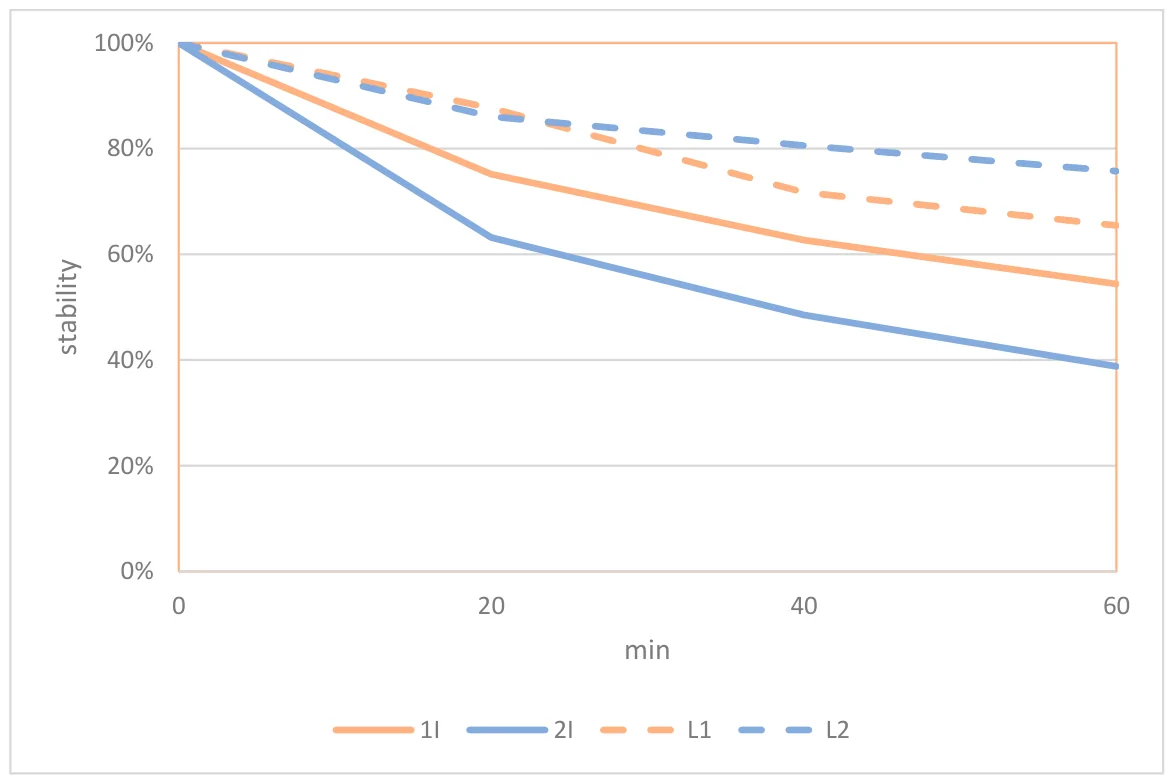
Photostability of **1I**, **2I**, **L1,** and **L2** irradiated with green LED light for 1 h.

As shown in Figure 3, both free BODIPY derivatives underwent marked photobleaching, retaining less than 50% of their initial absorbance after 1 h of continuous illumination. In contrast, the liposome-encapsulated formulations exhibited significantly enhanced photostability, retaining more than 65% of their initial absorbance after the same irradiation period.

Furthermore, no appreciable changes in the UV-Vis absorption profiles were observed for either the free or liposome-encapsulated BODIPYs throughout the irradiation period. In particular, no additional absorption bands or significant spectral shifts were detected, indicating that photobleaching occurred without the accumulation of stable photoproducts or other spectroscopically distinguishable species under the experimental conditions employed.

The apparent singlet oxygen (^1^O_2_) generation efficiency of the synthesized BODIPY derivatives was evaluated by monitoring the photooxidative degradation of 1,3-diphenylisobenzofuran (DPBF) under green LED irradiation for 10 min. Relative ^1^O_2_ generation efficiencies were determined by comparing the DPBF photobleaching kinetics of each photosensitizer with that of Rose Bengal, a well-established singlet oxygen photosensitizer used as the reference standard [61].

As expected, the two iodinated BODIPY derivatives exhibited comparable ^1^O_2_ generation efficiencies, with relative values of 1.16 and 1.13 for **1I** and **2I**, respectively. Following liposomal incorporation, the apparent ^1^O_2_ generation efficiency decreased to 0.71 for **L1** and 0.63 for **L2**.

### 3.2. In Vitro Studies

#### 3.2.1. Cellular Uptake

**Figure 4:**
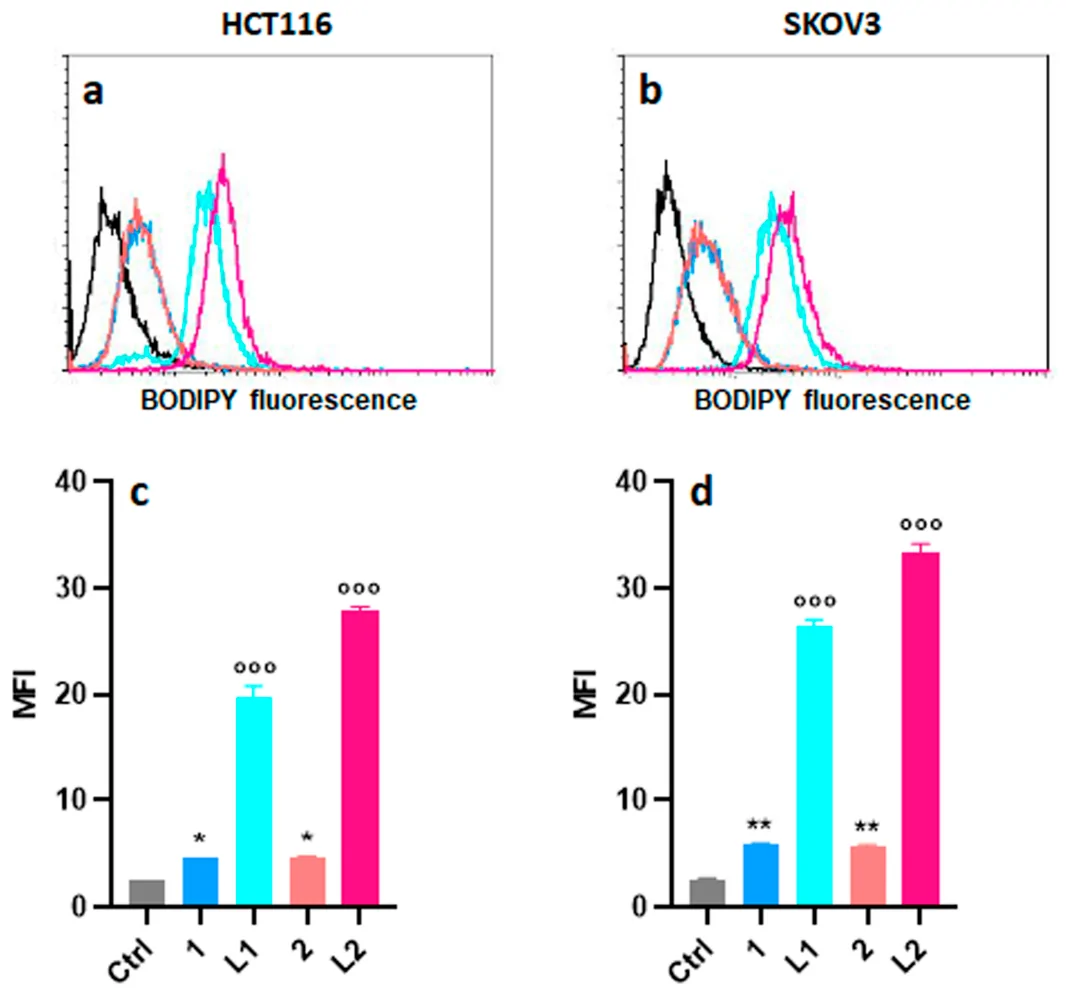
Intracellular uptake of **1**, **L1**, **2**, and **L2**. Representative flow cytometry plots (**a**,**b**) and quantitative analysis of 3 independent experiments (**c**,**d**), obtained in HCT116 and SKOV3 cells, are reported (mean ± S.D. of 3 independent experiments, each including at least 3 biological replicates per sample; * *p* < 0.05; ** *p* < 0.01 vs. Ctrl; °°° *p* < 0.001 vs. Ctrl and free BODIPY).

The intracellular uptake of **1** and **2** and liposome-incorporated compounds was evaluated by flow cytometry, exploiting the intrinsic fluorescence of the two non-iodinated BODIPY derivatives. Preliminary analyses revealed that cells treated with unloaded liposomes exhibited low median fluorescence intensity (MFI) values comparable to those observed in untreated control (Ctrl) cells in both cell lines. Representative flow cytometry histograms for each cell line as well as the corresponding quantitative analysis summarizing all independent experiments are presented in Figure 4.

In both cell lines, treatment with either free PSs or their liposome-loaded counterparts resulted in a marked rightward shift in the fluorescence signal (Figure 4a,b), indicative of enhanced intracellular accumulation of BODIPY compounds. Importantly, the increase in fluorescence intensity was significantly greater for both liposomal formulations than for the corresponding free BODIPYs, demonstrating the ability of liposomal delivery to improve cellular uptake. Furthermore, the uptake of **L1** and **L2** was more pronounced in SKOV3 cells than in HCT116 cells.

**Figure 5:**
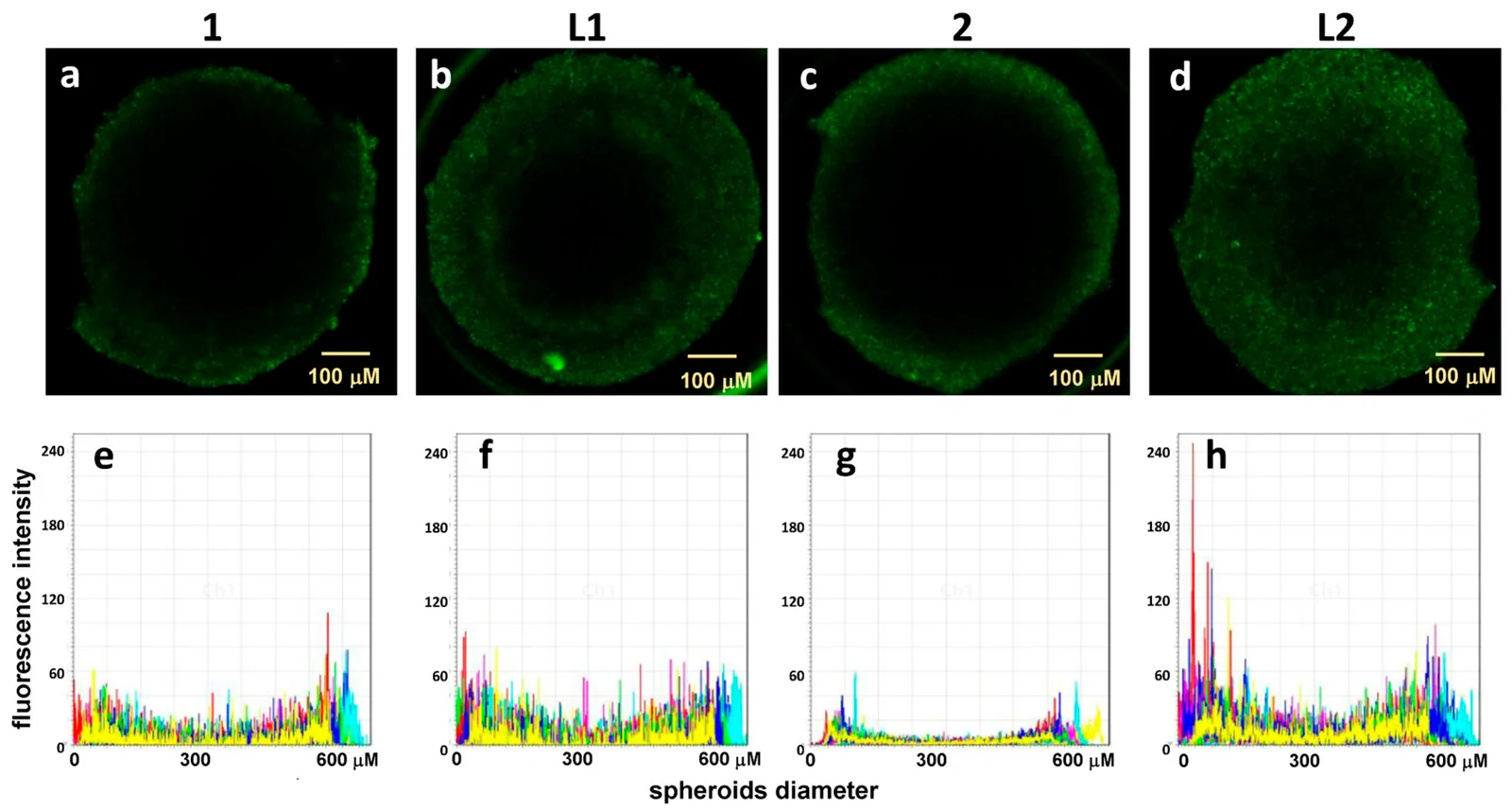
Penetration of **1**, **L1**, **2,** and **L2** (100 nM) in HCT116 spheroids after 24 h of incubation. Representative fluorescence images and quantitative analysis from three independent experiments are shown. Images show BODIPY fluorescence at the equatorial plane of the spheroids (**a**–**d**), while histograms represent the analysis of distribution and intensity of the fluorescence measured along 12 randomly selected diameters, traced across the equatorial plane of each spheroid, with each diameter represented by a different color (**e**–**h**).

Confocal microscopy analysis performed on HCT116 multicellular spheroids after 24 h of incubation with **1**, **L1**, **2,** and **L2** further supported these findings (Figure 5). Examination of fluorescence distribution and intensity profiles across the equatorial planes of the spheroids (Figure 5a–d) revealed a higher accumulation and deeper penetration of the liposomal formulation compared with the free compound. These results indicate that liposomal incorporation enhances photosensitizer uptake not only in conventional two-dimensional cultures but also in three-dimensional tumor models. Interestingly, penetration of **L1** and **L2** was not limited to the external cell rim of the spheroids; fluorescence was also detected in the deeper inner layers, suggesting a more extensive penetration throughout the three-dimensional structure for both liposomal formulations.

In light of these results, all subsequent studies were focused exclusively on the two liposomal nanoformulations, owing to their superior intracellular accumulation relative to the corresponding free photosensitizers.

#### 3.2.2. Effect on Cell Viability

To evaluate the photodynamic efficacy of **L1** and **L2**, HCT116 and SKOV3 cells were exposed to increasing concentrations of the two formulations, irradiated for 1 h, and subsequently incubated for 24 h in drug-free medium before cell viability determination by the MTT assay. Under these conditions, both formulations exhibited potent cytotoxic activity, with IC_50_ values in the nanomolar range (Table 2).

No intrinsic cytotoxicity was detected in the absence of light activation (dark conditions), even when the PS concentration was increased to 500 nM, and the irradiation step was omitted from the treatment protocol.

To exclude any contribution of the carrier system to the observed biological effects, the cytotoxicity of empty liposomes was also evaluated. HCT116 and SKOV3 cells were treated with liposome dilutions corresponding to those employed for **L1** and **L2**, and the resulting IC_50_ values, expressed as BODIPY-equivalent concentrations, exceeded 500 nM in all cases, indicating negligible carrier-associated toxicity.

No statistically significant differences in photodynamic potency were observed between the two formulations in either cell line. However, SKOV3 cells displayed significantly greater sensitivity to treatment than HCT116 cells, with IC_50_ values approximately 2.5-fold and 2-fold lower following exposure to **L1** and **L2**, respectively.

To assess the long-term reproductive capacity of cells after PDT with **L1** and **L2**, clonogenic assays were also performed in HCT116 cells. This assay represents a more stringent endpoint than short-term viability measurements, as it evaluates the ability of surviving cells to retain their proliferative potential and generate colonies after photodynamic damage. Clonogenic experiments were carried out exclusively in HCT116 cells because, under our experimental conditions, SKOV3 cells did not consistently form colonies and were therefore not suitable for this analysis.

**Figure 6:**
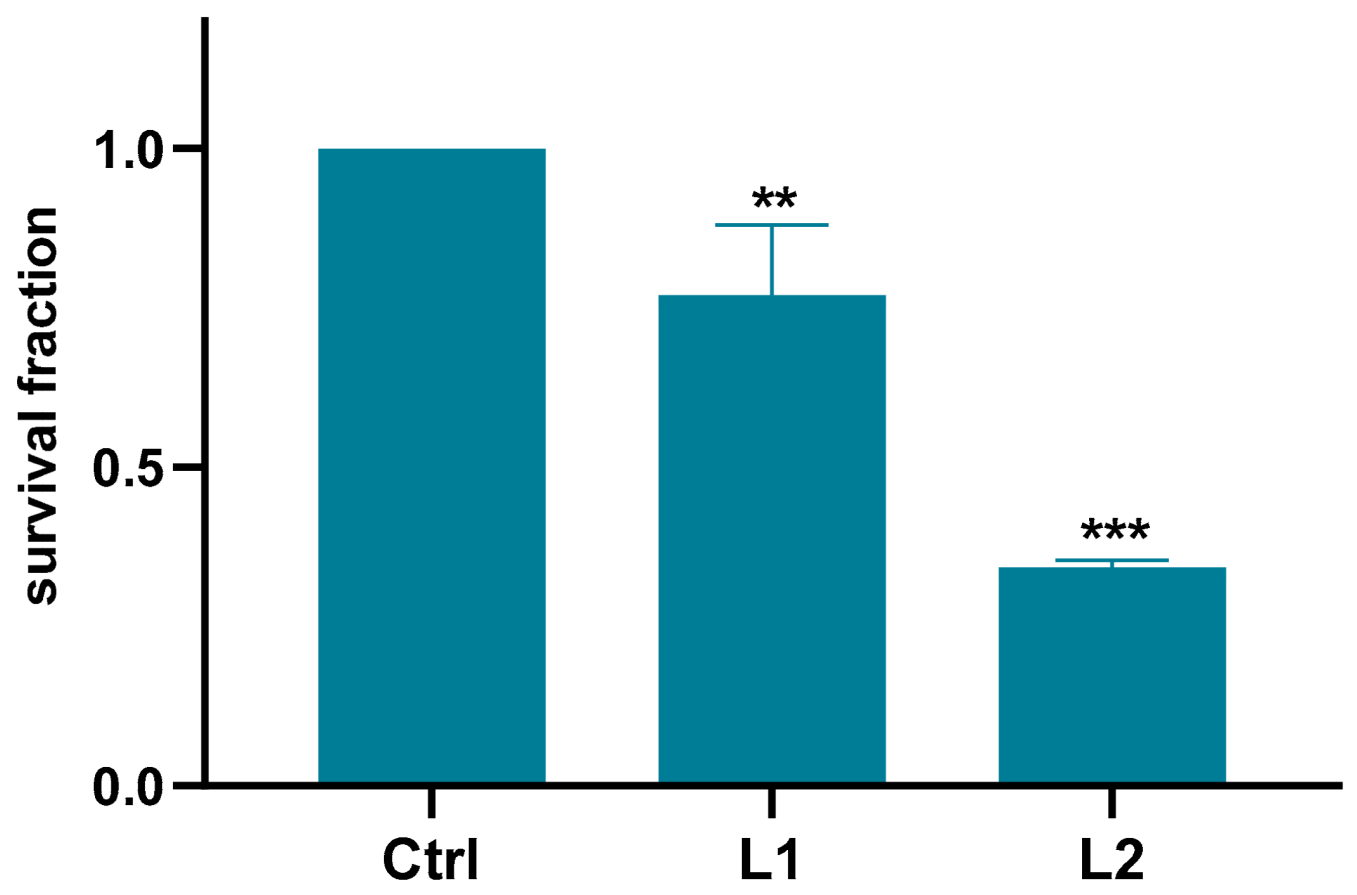
Survival fractions obtained following clonogenic assay in HCT116 cells treated with **L1** and **L2** at concentrations corresponding to the MTT-derived IC_50_ values as described (mean ± S.D. of 3 independent experiments, each including at least 3 biological replicates per sample; ** *p* < 0.01 and *** *p* < 0.001 vs. Ctrl).

Figure 6 shows that both compounds significantly reduced the surviving fraction compared with untreated control cells, indicating a marked impairment of the long-term proliferative capacity. In particular, treatment with **L1** resulted in a moderate but significant decrease in colony-forming ability (approximately 75% survival), whereas **L2** produced a much stronger effect, reducing the surviving fraction to approximately 35% of control levels (Figure 6). Thus, even when administered at concentrations corresponding to their MTT-derived IC_50_ values, the two BODIPY derivatives differ in their ability to suppress clonogenic survival, with **L2** exhibiting a substantially greater long-term cytotoxic effect than **L1**.

**Figure 7:**
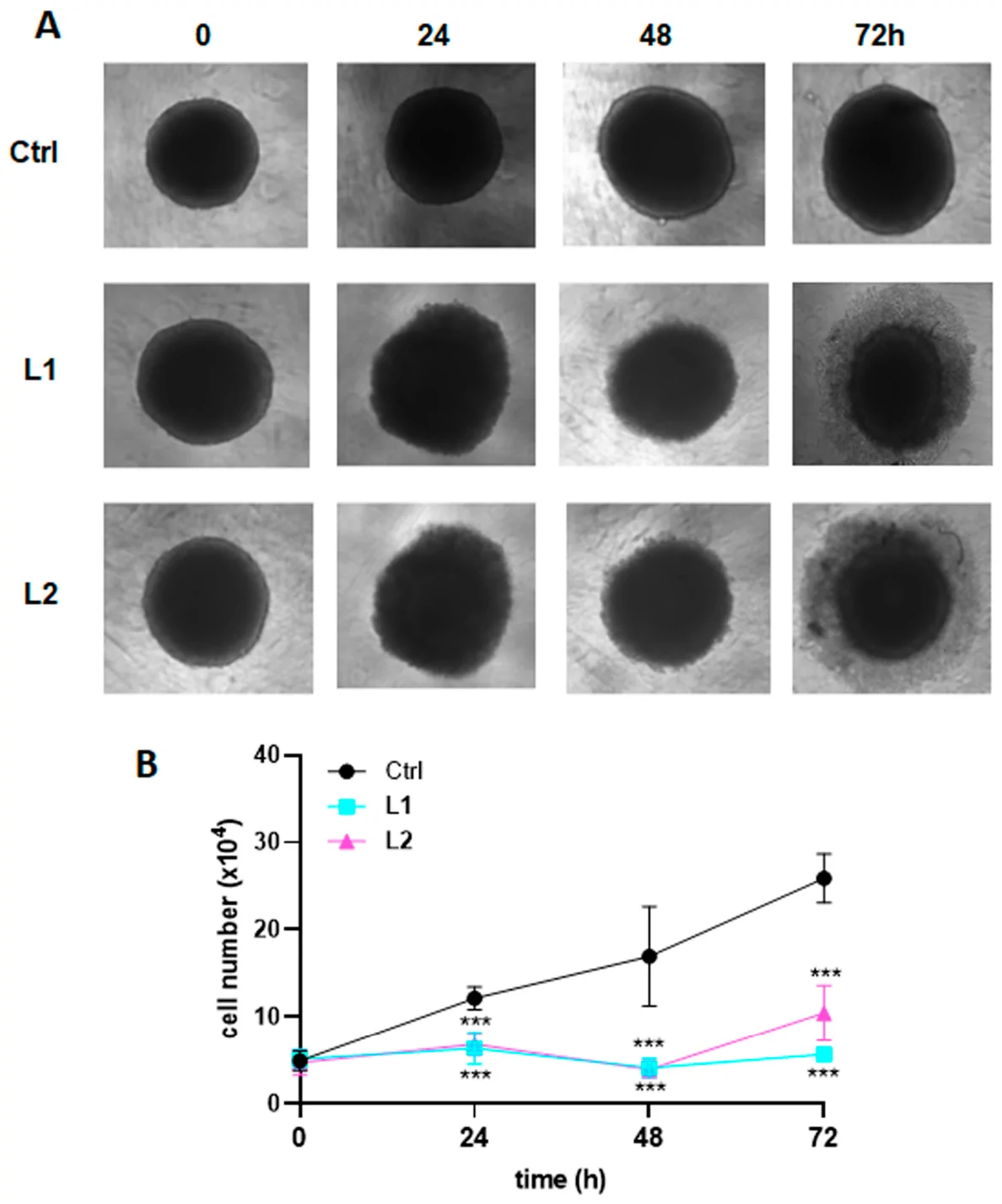
HCT116 spheroid images 10x magnification, (**A**) and growth curves (**B**) following 24 h of treatment with **L1** and **L2**, 1 h of irradiation, and incubation in drug-free medium in the dark. Pictures of spheroids (**A**) and count of viable cells (**B**) were performed immediately after irradiation (time 0) and 24, 48, and 72 h later (mean ± S.D. of 3 independent experiments, each including 3/5 biological replicates (i.e., spheroids) per sample; *** *p* < 0.001 vs. Ctrl).

Three-dimensional multicellular spheroids obtained from HCT116 cells were used to evaluate the photodynamic efficacy of **L1** and **L2** in a model that better recapitulates the architecture and diffusion limitations of solid tumors than conventional two-dimensional cultures. Spheroid growth kinetics were monitored for 72 h following treatment and photoactivation. Representative images were acquired immediately after irradiation (0) and at 24, 48, and 72 h post-irradiation using a camera connected to an Olympus IX81 microscope. At each time point, 3/5 spheroids per experimental condition were collected, enzymatically disaggregated, and the number of viable cells was determined by Trypan blue exclusion to generate growth curves (Figure 7).

This analysis was performed exclusively using HCT116 spheroids, as SKOV3 cells failed to form stable and reproducible spheroids under our experimental conditions, making them unsuitable for three-dimensional studies.

As shown in the exemplificative images in Figure 7A, untreated HCT116 spheroids exhibited a progressive increase in size over the 72 h observation period. In contrast, PDT with either **L1** or **L2** markedly inhibited spheroid growth, and this suppressive effect persisted for the entire experimental timeframe, indicating sustained photodynamic activity in the three-dimensional model. In addition to growth inhibition, treated spheroids underwent progressive structural deterioration, characterized by the detachment and shedding of cells from the outer layers. This phenomenon became particularly evident 72 h after irradiation, indicating extensive disruption of spheroid integrity.

Quantitative analysis based on the Trypan blue-exclusion assay corroborated the morphological findings. Untreated HCT116 spheroids displayed a time-dependent increase in the number of viable cells, consistent with continued spheroid growth. In contrast, PDT with both photosensitizers significantly reduced the viable cell population compared with untreated controls, with statistically significant differences observed at all examined time points (Figure 7B).

#### 3.2.3. ROS Production

**Figure 8:**
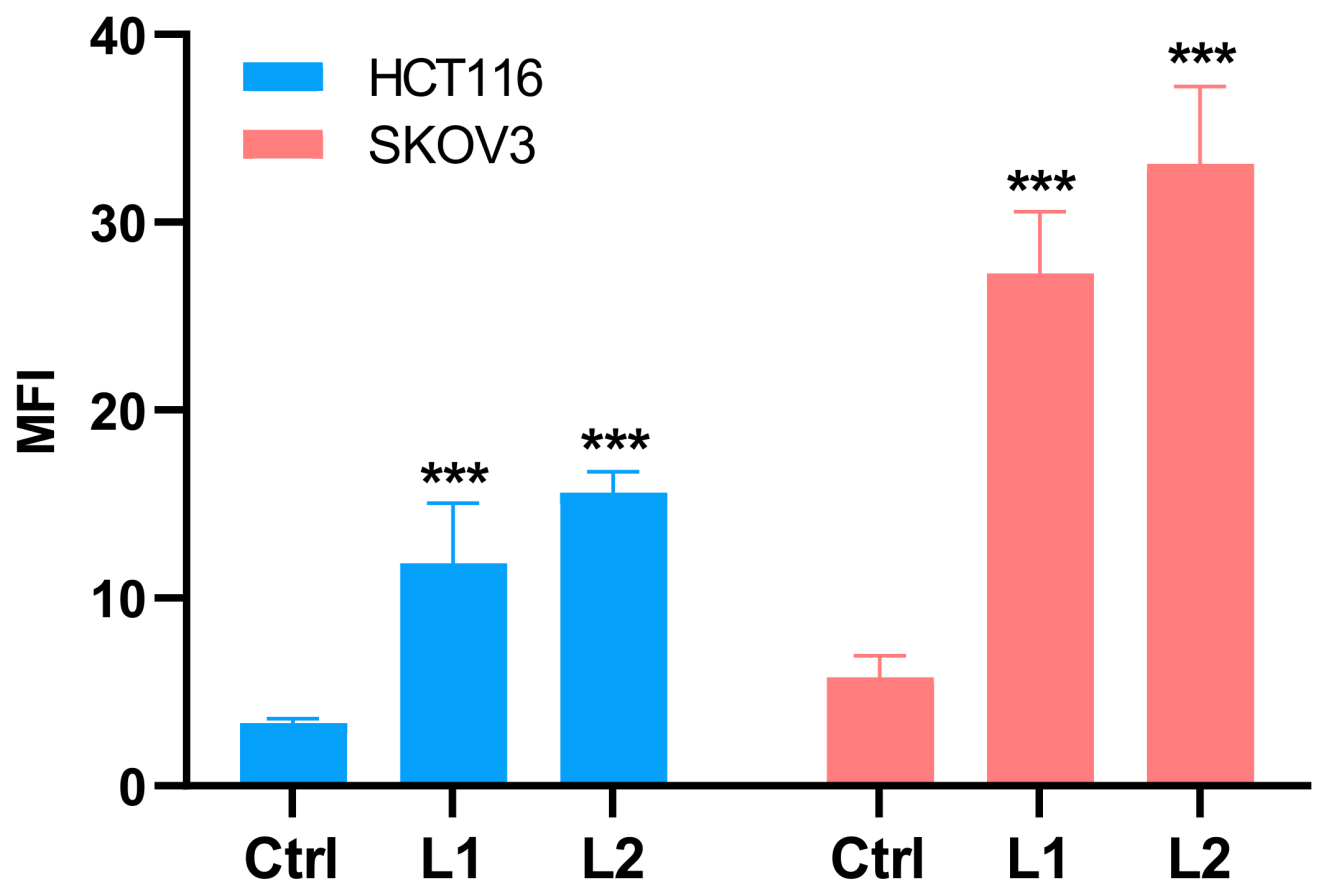
ROS levels in HCT116 and SKOV3 cell lines treated with **L1** and **L2** for 24 h at equitoxic concentrations corresponding to their respective IC_50_ values, incubated with H2DCF-DA, irradiated for 5 min, and analyzed by flow cytometry (mean ± S.D. of 3 independent experiments, each including at least 3 biological replicates per sample; *** *p* < 0.001 vs. Ctrl).

Figure 8 shows intracellular ROS generation assessed using the fluorescent probe H2DCF-DA. A significant increase in ROS production was detected following 24 h of treatment with **L1** and **L2** in both HCT116 and SKOV3 cell lines. Consistent with the cell viability data, exposure to the photosensitizers resulted in markedly enhanced ROS production compared with untreated controls in both cellular models.

#### 3.2.4. Cell Death Induction

The ability of **L1** and **L2** to induce the most common types of cell death modalities, including apoptosis, necrosis, necroptosis, and ferroptosis, was investigated.

**Figure 9:**
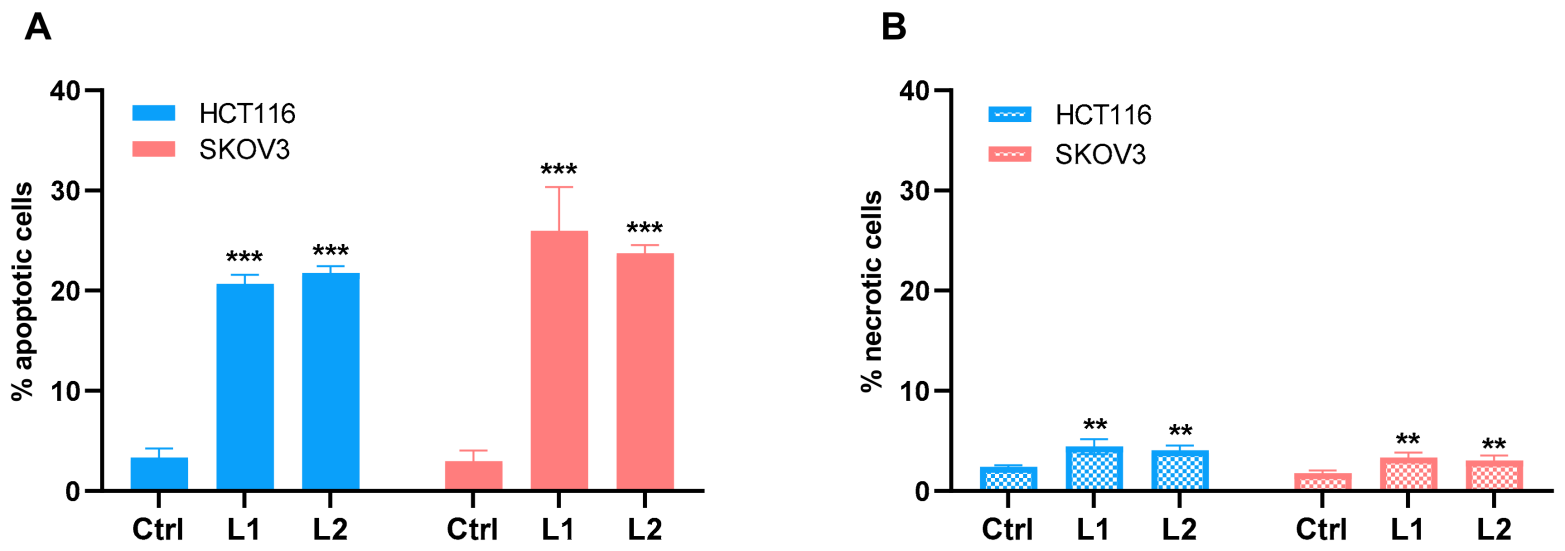
Percentages of apoptotic (**A**) and necrotic (**B**) HCT116 and SKOV3 cells following 24 h of treatment with **L1** and **L2** at equitoxic concentrations corresponding to the respective IC_50_ values and 1 h of irradiation. Incubation for 24 h in drug-free medium. Propidium iodide was used as a DNA probe (mean ± SE of 3 independent experiments, each including at least 3 biological replicates per sample; ** *p* < 0.01 and *** *p* < 0.001 vs. Ctrl).

Apoptotic and necrotic cell death were first evaluated in HCT116 and SKOV3 cells treated with equitoxic concentrations of the two photosensitizers, corresponding to their respective IC_50_ values. PDT induced a marked and significant increase in apoptotic cell death in both cell lines, irrespective of the photosensitizer employed. In contrast, only a modest, although statistically significant, increase in the percentage of necrotic cells was observed, indicating that apoptosis represents the predominant mode of cell death triggered by PDT under the experimental conditions used (Figure 9).

**Figure 10:**
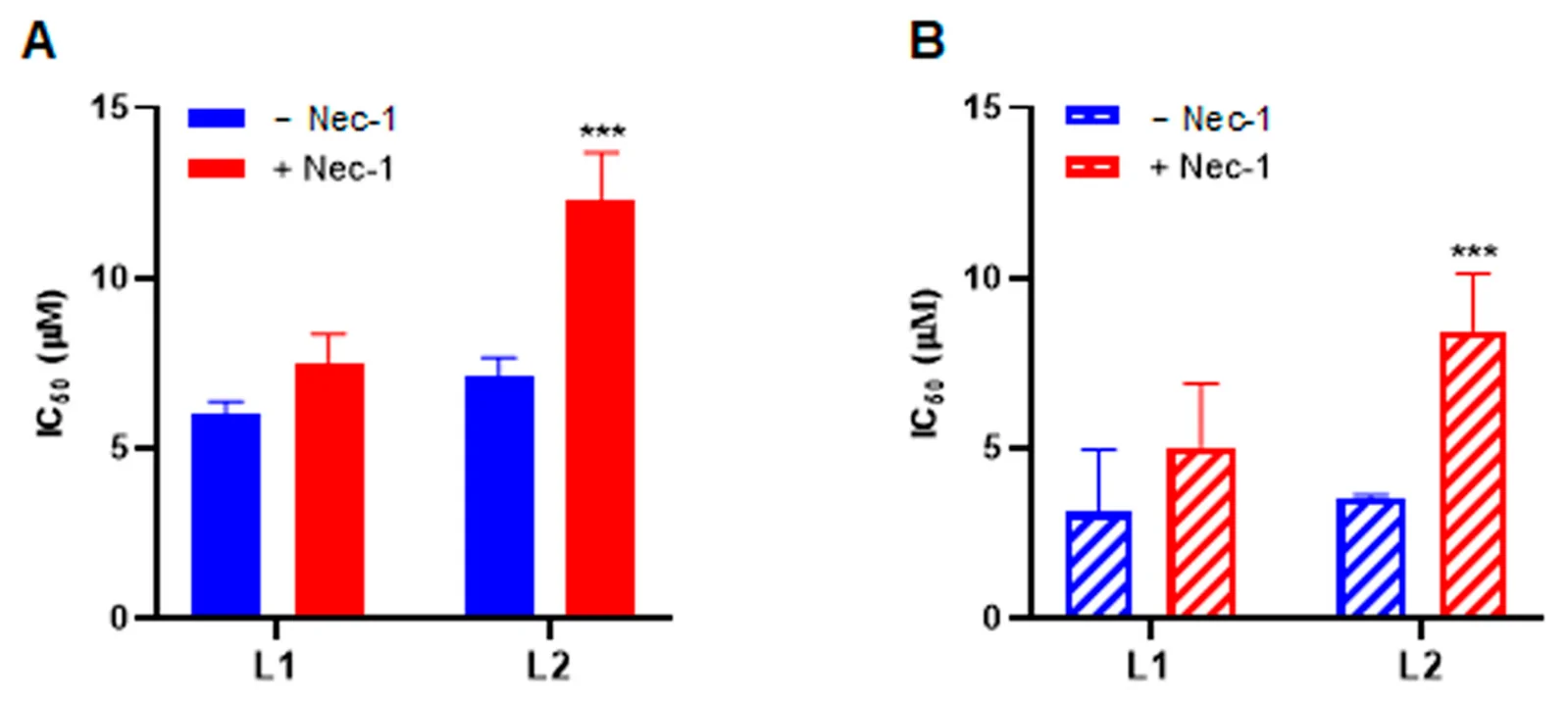
IC_50_ values obtained following 24 h of incubation of HCT116 (**A**) and SKOV3 (**B**) cells with the PSs in the presence or absence of 20 μM Nec-1, 1 h of irradiation under green LED light, 24 h of incubation in Nec-1-containing medium, and MTT assay (mean ± S.D. of 3/5 independent experiments; *** *p* < 0.05 vs. HCT116).

To further elucidate the contribution of alternative regulated cell death pathways, the involvement of necroptosis was investigated by determining the IC_50_ values of both photosensitizers in the presence or absence of Necrostatin-1 (Nec-1), a selective inhibitor of RIPK1-mediated necroptosis. The addition of Nec-1 led to a moderate increase in IC_50_ values for either **L1** or **L2** in both HCT116 and SKOV3 cell lines, although the extent of this effect depended on both the photosensitizer and the cellular model (Figure 10). In particular, a statistically significant increase in IC_50_ values was observed only for **L2** in both cell lines, suggesting a partial involvement of RIPK1-dependent signaling pathways in the response to PDT. However, the magnitude of these changes was relatively modest, indicating that necroptosis plays only a minor role in the overall photodynamic cytotoxicity induced by the investigated PSs.

**Figure 11:**
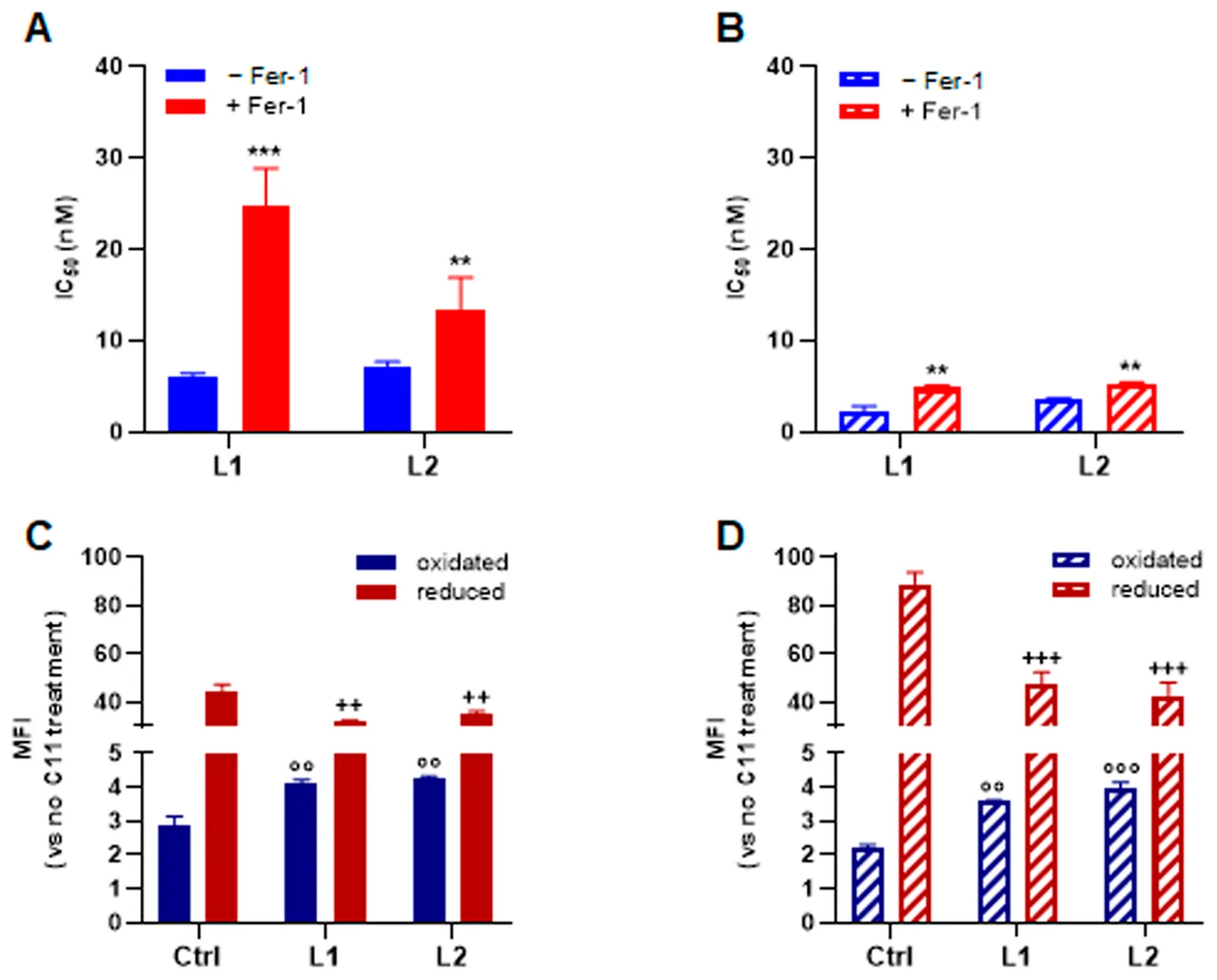
Effect of Fer-1 treatment on HCT116 and SKOV3 cell viability (**A**,**B**) and lipid peroxidation evaluation (**C**,**D**) following PDT (mean ± S.D. of 3 independent experiments; ** *p* < 0.01 and *** *p* < 0.001 vs. -Fer-1; °° *p* < 0.01 and °°° *p* < 0.001 vs. Ctrl (oxidated BODIPY 581/591 C11 form); ++ *p* < 0.01 and +++ *p* < 0.001 vs. Ctrl (reduced BODIPY 581/591 C11 form)).

Given the central role of oxidative stress in photodynamic therapy and the limited contribution of necroptosis, we next explored the possible involvement of ferroptosis, using both pharmacological and biochemical approaches. To assess the functional contribution of ferroptosis, cells were treated with the ferroptosis inhibitor Ferrostatin-1 (Fer-1) before PDT. As shown in Figure 11, Fer-1 treatment increased HCT116 and SKOV3 cell viability following PDT, resulting in higher IC_50_ values compared with PDT alone. This protective effect was particularly evident in the first cell line, whereas a more modest increase was observed in the second one, indicating that ferroptosis contributes, at least in part, to PDT-mediated cytotoxicity.

To further substantiate these findings, lipid peroxidation was evaluated using BODIPY™ 581/591 C11 staining. PDT induced a marked increase in the oxidized form of the probe together with a corresponding decrease in the reduced fraction (Figure 11C,D), demonstrating enhanced lipid peroxidation following treatment. This effect was detected in both cellular models, although with different magnitudes.

## 4. Discussion

Efficient intracellular delivery of photosensitizers is a key determinant of PDT efficacy, as limited cellular uptake and suboptimal intracellular distribution can markedly compromise therapeutic outcomes [7,8,10]. The clinical translation of many PSs is further hampered by poor aqueous solubility, low bioavailability, and aggregation in biological media [4,16]. Nanocarrier-based formulations, particularly liposomes, have therefore emerged as a promising strategy to overcome these limitations by improving photosensitizer stability, dispersion, and intracellular delivery, ultimately enhancing photodynamic efficacy [62,63].

The present study aimed to synthesize two novel BODIPY-based photosensitizers, incorporate them in liposomes, and evaluate the physicochemical properties of the resulting formulations, as well as their photodynamic activity, in two human tumor cell lines. From a physicochemical perspective, the absence of significant H- or J-type aggregation, evidenced by UV-Vis spectroscopy, represents a favorable feature, as aggregation compromises the photophysical performance of many photosensitizers [64,65]. This behavior is likely attributable to the molecular design of the BODIPY derivatives, in which steric hindrance generated by the four methyl substituents and the nearly perpendicular orientation of the *meso*-phenyl ring limits intermolecular π-stacking, thereby preserving the monomeric state of the chromophores. Consistent with their closely related structures, the two iodinated BODIPY derivatives exhibited comparable ^1^O_2_ generation efficiencies (1.16 and 1.13 for **1I** and **2I**, respectively), indicating that the different position of the methoxycarbonyl substituent has a negligible impact on the photophysical properties of the BODIPY core. Following liposomal incorporation, the apparent ^1^O_2_ generation efficiency decreased (0.71 for **L1** and 0.63 for **L2**), as expected for membrane-confined photosensitizers [66]. Because of the short lifetime and diffusion distance of ^1^O_2_, a fraction of the generated species is likely consumed close to its formation site within or near the lipid bilayer before it can diffuse to the DPBF probe, leading to an underestimation of the apparent ^1^O_2_ yield [67,68,69]. This interpretation is consistent with the diffusion-controlled nature of the DPBF assay, whose signal depends not only on the amount of ^1^O_2_ generated but also on its accessibility to the probe [67]. In addition, the lipid bilayer modifies the local photophysical environment of the photosensitizer. Changes in oxygen partitioning, membrane organization, and sensitizer-lipid interactions may influence excited-state deactivation pathways and the apparent efficiency of ^1^O_2_ production [70]. Furthermore, locally generated ^1^O_2_ may react with nearby unsaturated lipids before diffusion into the surrounding medium, thereby reducing the amount detected by bulk-solution probes [70,71]. This interpretation is consistent with reports that BODIPY photosensitizers can embed in lipid membranes and retain strong photodynamic function in membrane-associated settings [27,72]. Since triplet-state lifetime, oxygen distribution within the membrane, and intersystem crossing efficiency were not directly measured here, the relative contribution of these mechanisms cannot be resolved from the present data.

Importantly, the moderate reduction in apparent ^1^O_2_ generation did not translate into diminished biological activity. The high photodynamic efficacy observed is likely the result of multiple complementary mechanisms. Besides improving cellular uptake, liposomes may alter intracellular trafficking and subcellular localization, thereby promoting ROS-mediated damage in critical organelles [73,74]. Encapsulation may also reduce photosensitizer aggregation and self-quenching after cellular delivery, while the lipid microenvironment may influence oxygen availability and ROS reactivity [75,76]. In addition, a contribution of oxygen-independent Type I photochemical processes cannot be excluded [77,78]. Although we reported improved photostability and limited aggregation of the PS and increased cellular ROS production, the present data do not yet distinguish the relative contribution of all mechanisms involved. Besides generating cytotoxic ROS, photoexcited photosensitizers may undergo photooxidative degradation (photobleaching), a process that progressively reduces the amount of active photosensitizer available during irradiation [79]. Although photobleaching is generally considered detrimental when excessive, its extent also reflects the interaction between the photosensitizer and the reactive oxygen species generated during PDT [80]. In agreement with previous reports on iodinated BODIPY derivatives, the free compounds underwent measurable photobleaching upon irradiation [46,81]. Interestingly, liposomal incorporation markedly attenuated this process, suggesting that the lipid bilayer provides a protective microenvironment that limits photooxidative degradation while preserving efficient ROS generation [21,82].

Efficient intracellular delivery remains a major determinant of PDT efficacy, as the short lifetime and diffusion distance of reactive oxygen species confine photodynamic damage to the immediate vicinity of the photosensitizer [6,7,8]. In this context, the enhanced cellular uptake of the liposomal formulations compared with the free compounds, demonstrated in both HCT116 and SKOV3 cell lines, can be rationalized by the physicochemical properties of the nanosystem. The presence of the PEG-2000-PE coating makes direct membrane fusion unlikely, since the steric barrier imposed by the polyethylene glycol chains prevents close apposition between the liposomal and cellular bilayers [83,84]. Endocytosis therefore represents the most plausible internalization mechanism, consistent with the well-established uptake behavior of PEGylated nanoparticles [85]. However, as the uptake pathways were not experimentally investigated in the present study, this interpretation remains speculative. Additional support comes from confocal microscopy of HCT116 multicellular spheroids, which showed that **L1** and **L2** penetrated beyond the peripheral cell layers and accumulated throughout the spheroid volume. Given the highly hydrophobic nature of the BODIPY derivatives, spontaneous release into the aqueous extracellular environment is thermodynamically unfavorable, suggesting that the liposomes diffuse through the spheroid predominantly as intact nanoparticles, with cargo release occurring only after cellular internalization. Collectively, these findings indicate that liposomal incorporation effectively enhances photosensitizer delivery to poorly accessible tumor regions, where limited drug penetration and hypoxia often compromise PDT efficacy.

The therapeutic potential of a photosensitizer relies on its ability to selectively induce cytotoxicity upon light activation while remaining biologically inert in the absence of irradiation [5,65]. Because photodynamic damage may result in either transient metabolic impairment or irreversible loss of proliferative capacity, multiple complementary assays are required to accurately assess treatment efficacy [86,87,88]. Accordingly, both MTT and clonogenic assays were employed in the present study. While the MTT assay reflects short-term metabolic activity following treatment, the clonogenic assay evaluates the long-term ability of surviving cells to proliferate and form colonies, representing a more stringent indicator of irreversible cytotoxicity [89,90]. The combined use of these complementary approaches therefore provides a comprehensive evaluation of photodynamic efficacy by assessing both the immediate cellular response and the sustained suppression of tumor cell proliferation.

In agreement with this requirement, both liposomal formulations displayed potent photodynamic activity, with IC_50_ values in the low nanomolar range and no detectable dark toxicity up to the highest concentration tested. The absence of intrinsic cytotoxicity, together with the negligible toxicity of empty liposomes, demonstrates that the observed biological effects derive exclusively from photoactivation of the encapsulated BODIPY derivatives rather than from carrier-related toxicity. Notably, although the two formulations exhibited comparable photodynamic potency, SKOV3 cells were consistently more sensitive than HCT116 cells. This differential response suggests that factors beyond photosensitizer uptake, including variations in antioxidant capacity, intracellular ROS detoxification, DNA damage repair, or susceptibility to oxidative stress, may critically influence PDT efficacy [2,64,65]. Such cell type-dependent differences are well recognized as important determinants of photodynamic response and may contribute to the distinct sensitivity observed in the two tumor models.

Clonogenic assays further demonstrated that both formulations profoundly impaired the long-term reproductive capacity of HCT116 cells, the only cell line able to form colonies under our experimental conditions, indicating that PDT-induced damage extends well beyond the transient metabolic alterations detected by the MTT assay. Interestingly, despite exhibiting similar IC_50_ values in short-term viability assays, **L2** produced a markedly greater reduction in colony-forming ability than **L1**. This apparent discrepancy highlights the complementary information provided by clonogenic analysis and suggests that the two formulations may elicit qualitatively different biological responses. Specifically, **L2** may induce more extensive or less readily repairable cellular damage, ultimately compromising the ability of surviving cells to resume sustained proliferation even when their short-term metabolic activity remains relatively preserved.

Three-dimensional tumor spheroids provide a more physiologically relevant model than conventional monolayer cultures by reproducing cell–cell interactions, diffusion gradients, and the limited drug penetration typical of solid tumors [29,30]. Despite these barriers, both liposomal formulations effectively suppressed spheroid growth throughout the observation period and induced progressive disruption of spheroid architecture. These findings are fully consistent with the confocal microscopy data demonstrating nanoparticle penetration beyond the peripheral cell layers, indicating that the liposomal carrier effectively delivers the photosensitizer throughout the tumor mass. The maintenance of photodynamic activity in this more challenging model highlights the potential of the formulation to overcome one of the major limitations of PDT in solid tumors.

As expected, the pronounced photodynamic activity of both formulations was accompanied by a marked increase in intracellular ROS generation following irradiation. Since ROS are the primary mediators of PDT-induced cytotoxicity, these findings confirm that liposomal incorporation preserves the ability of the BODIPY derivatives to efficiently trigger oxidative stress inside tumor cells.

The biological consequences of ROS generation largely depend on their intracellular localization and intensity, which determine the activation of distinct regulated cell death pathways [5,88,91]. In agreement with the canonical mechanism of PDT, apoptosis emerged as the predominant mode of cell death induced by both formulations, whereas necrosis contributed only marginally. Pharmacological inhibition studies further indicated that necroptosis plays a limited role, as Necrostatin-1 produced only modest increases in IC_50_ values, reaching statistical significance exclusively for **L2**. Conversely, the partial rescue afforded by Ferrostatin-1, together with the marked increase in lipid peroxidation detected by BODIPY™ 581/591 C11, supports the involvement of ferroptosis in the photodynamic response. These findings are consistent with increasing evidence that lipid peroxidation is a major downstream consequence of PDT and support a cooperative contribution of apoptosis and ferroptosis to the cytotoxic activity of the formulations [92,93,94].

Taken together, the present findings demonstrate that the rational molecular design of iodinated BODIPY photosensitizers, combined with liposomal formulation, effectively enhances their delivery and photodynamic performance. Nevertheless, despite these promising features, clinically established PSs currently benefit from decades of preclinical and clinical validation. Therefore, the favorable physicochemical characteristics of BODIPY derivatives should be considered as a promising platform whose translational potential remains to be confirmed through comprehensive in vivo and clinical studies.

## 5. Conclusions

In conclusion, this study demonstrates that liposomal incorporation is an effective strategy to enhance the pharmaceutical and biological performance of iodinated BODIPY photosensitizers. The developed formulations exhibited favorable physicochemical properties, high incorporation efficiency, preserved photoactivity, improved photostability, and enhanced cellular uptake, ultimately resulting in potent photodynamic activity at nanomolar concentrations with negligible dark toxicity. Importantly, the formulations retained their efficacy in 3D tumor spheroids, supporting their ability to overcome diffusion barriers that typically limit the effectiveness of PDT in solid tumors.

Beyond their pronounced antitumor activity, mechanistic investigations revealed that the photodynamic response is primarily mediated by apoptosis, with ferroptosis also contributing to the overall cytotoxic effect through ROS-induced lipid peroxidation. These findings further expand the understanding of the biological mechanisms triggered by BODIPY-based photosensitizers and highlight the potential advantages of liposomal delivery in modulating their intracellular activity.

Nevertheless, the present study has some limitations. The biological evaluation was restricted to in vitro monolayer cultures and multicellular tumor spheroids, and the proposed mechanism of liposomal internalization was inferred from the physicochemical characteristics of the formulations rather than experimentally demonstrated. In addition, some relevant physicochemical properties of the BODIPY derivatives were not specifically investigated.

Despite these limitations, the study provides a comprehensive characterization of the photodynamic activity of the liposomal BODIPY formulations, demonstrating their antitumor efficacy and offering mechanistic insights into the cellular responses involved. Overall, the integration of rational photosensitizer design with liposomal nanotechnology provides a promising platform for improving PDT performance. Future studies should therefore focus on elucidating the cellular uptake mechanisms, evaluating therapeutic efficacy and safety in appropriate animal tumor models, and assessing pharmacokinetic behavior and tumor accumulation to support clinical translation. Despite these limitations, the present work establishes a robust proof of concept for the development of BODIPY-based liposomal formulations as effective nanomedicines for photodynamic cancer therapy.

## Figures and Tables

**Table 1:** Physicochemical properties of the formulations assessed by DLS.

Sample	Diameter (nm)	PDI	Zeta Potential (mV)
Liposomes	117.9	0.105	−12.7
Liposomes/**1I**	87.16	0.146	−12.7
Liposomes/**1**/**1I**	89.87	0.148	−10.5

**Table 2:** IC_50_ values obtained following 24 h of incubation of HCT116 and SKOV3 cells with the PSs, 1 h of irradiation under green LED light, 24 h of incubation in drug-free medium, and MTT assay (mean ± S.D. of 3/5 independent experiments; * *p* < 0.05 vs. HCT116).

IC_50_ (nM)	HCT116		SKOV3	
	Dark	Light	Dark	Light
**L1**	>500	6.0 ± 0.85	>500	2.36 ± 0.44 *
**L2**	>500	7.13 ± 0.53	>500	3.52 ± 0.11 *

## Data Availability

The original contributions presented in this study are included in the article. Further inquiries can be directed to the corresponding authors.
